# Expression Profile of Human Fc Receptors in Mucosal Tissue: Implications for Antibody-Dependent Cellular Effector Functions Targeting HIV-1 Transmission

**DOI:** 10.1371/journal.pone.0154656

**Published:** 2016-05-10

**Authors:** Hannah M. Cheeseman, Ann M. Carias, Abbey B. Evans, Natalia J. Olejniczak, Paul Ziprin, Deborah F. L. King, Thomas J. Hope, Robin J. Shattock

**Affiliations:** 1 Imperial College London, Department of Medicine, Section of Virology, Group of Mucosal Infection and Immunity, London, United Kingdom; 2 Northwestern University, Feinberg School of Medicine, Cell and Molecular Biology Department, Chicago, Illinois, United States of America; 3 Imperial College London, Department of Surgery, St. Mary’s Hospital, London, United Kingdom; Harvard Medical School, UNITED STATES

## Abstract

The majority of new Human Immunodeficiency Virus (HIV)-1 infections are acquired via sexual transmission at mucosal surfaces. Partial efficacy (31.2%) of the Thai RV144 HIV-1 vaccine trial has been correlated with Antibody-dependent Cellular Cytotoxicity (ADCC) mediated by non-neutralizing antibodies targeting the V1V2 region of the HIV-1 envelope. This has led to speculation that ADCC and other antibody-dependent cellular effector functions might provide an important defense against mucosal acquisition of HIV-1 infection. However, the ability of antibody-dependent cellular effector mechanisms to impact on early mucosal transmission events will depend on a variety of parameters including effector cell type, frequency, the class of Fc-Receptor (FcR) expressed, the number of FcR per cell and the glycoslyation pattern of the induced antibodies. In this study, we characterize and compare the frequency and phenotype of IgG (CD16 [FcγRIII], CD32 [FcγRII] and CD64 [FcγRI]) and IgA (CD89 [FcαR]) receptor expression on effector cells within male and female genital mucosal tissue, colorectal tissue and red blood cell-lysed whole blood. The frequency of FcR expression on CD14+ monocytic cells, myeloid dendritic cells and natural killer cells were similar across the three mucosal tissue compartments, but significantly lower when compared to the FcR expression profile of effector cells isolated from whole blood, with many cells negative for all FcRs. Of the three tissues tested, penile tissue had the highest percentage of FcR positive effector cells. Immunofluorescent staining was used to determine the location of CD14+, CD11c+ and CD56+ cells within the three mucosal tissues. We show that the majority of effector cells across the different mucosal locations reside within the subepithelial lamina propria. The potential implication of the observed FcR expression patterns on the effectiveness of FcR-dependent cellular effector functions to impact on the initial events in mucosal transmission and dissemination warrants further mechanistic studies.

## Introduction

The majority of new Human Immunodeficiency Virus (HIV-1) infections occur via sexual transmission at the mucosal portals of entry, specifically the male and female genital tracts and the rectal mucosa [1]. While it has been suggested that antibody-dependent cellular effector functions might have important defensive roles against pathogenic infections at mucosal surfaces, little is known about the phenotype and density of antibody effector cells found within these tissues.

The partial protective efficacy (31.2%) of the RV144 HIV-1 vaccine trial in Thailand [2] has driven an enhanced interest in the role of non-neutralizing antibodies in mucosal protection. Extensive correlates analysis of the RV144 trial identified that a reduced risk of HIV-1 acquisition was positively associated with the development of serum IgG antibodies (particularly IgG3) to the V1V2 region of the Env trimer able to mediate antibody-dependent cellular cytotoxicity (ADCC) [3–5]. This positive association was negated in the presence of high levels of IgA antibodies able to block Fc-gamma receptor (FcγR) mediated ADCC through competitive binding to V1V2 [4]. These observations have led to the suggestion that ADCC activity might be an important component of prophylactic vaccines against HIV-1 and potentially a mechanistic correlate of protection in the RV144 trial [3, 6–11].

Antibody-dependent cellular effector functions are triggered by the localized clustering of cell membrane Fc receptors (FcR) through binding to the Fc portion of complexed antibodies: in the case of HIV-1, opsonized (or antibody coated) infected cells and/or cells coated with opsonized viral particles [12]. ADCC is most efficiently triggered through antibody Fc engagement of CD16 (FcγRIII), predominantly found on the surface of natural killer (NK) cells, neutrophils, and subpopulations of monocytes, macrophages and dendritic cells (DC) [13–15]. Engagement of CD16 triggers the directional release across the lytic synapse of the content of cytotoxic granules and, in the case of NK cells, the expression of cell death-inducing molecules, resulting in death of the opsonized cells. ADCC can also be triggered by crosslinking of FcγRI (CD64) and FcγRII (CD32) on myeloid cells (monocytes, macrophages and dendritic cells), although the mechanism and efficiency of cell-mediated extracellular lysis remains controversial. However, other antibody-dependent effector functions, specifically antibody-dependent cellular phagocytosis (ADCP) and antibody-dependent cellular viral inhibition (ADCVI) may also impact on initial events in mucosal HIV-1 infection [16]. In contrast to ADCC, which for myeloid cells requires incubation times of up to 24h, ADCP is reported to occur rapidly and efficiently within 1–4h [14]. ADCP predominately acts through engagement of CD32 (FcγRII), CD64 (FcγRI) and CD89 (FcαR) on monocytes, macrophages, and dendritic cells, leading to phagocytosis of opsonized virus [17] and infected cells and their subsequent degradation [18]. ADCVI can be mediated by ADCC, ADCP, and the release of HIV inhibitory β-chemokines blocking the onward infection of susceptible cells, and is a functional readout of the sum of these antibody-mediated effector mechanisms [19–21].

In terms of vaccine design, it is important to determine the phenotype and frequency of antibody effector cells present within the mucosal portals of HIV-1 transmission. Natural Killer (NK) cells, Myeloid dendritic cells (mDC) and cells of a monocyte lineage (CD14+) are the predominant cells involved in Fc effector functions. Research conducted in the 1990s used RNA and histological techniques to demonstrate expression of CD16 (FcγRIII) and CD32 (FcγRII) on colorectal and cervical mucosa [22–25]. More recently, studies have used flow cytometry to attempt to further characterize immune cells within cervical tissue [25, 26]. However, there have been no comprehensive studies to characterize and directly compare immune cells from different mucosal sites associated with the sexual transmission of HIV-1.

The ability of antibody-dependent cellular effector mechanisms to impact on early mucosal transmission events will depend on a variety of parameters including effector cell type frequency, the class of FcR expressed, the number of FcR per cell, and the glycoslyation pattern of the induced antibodies. Therefore, in this study we sought to determine the frequency and phenotype of IgG (CD16 [FcγRIII], CD32 [FcγRII] and CD64 [FcγRI]) and IgA (CD89 [FcαR]) receptor expression on effector cells within penile glans, ectocervical and colorectal tissue using tissue digestion and multicoloured flow cytometry techniques. The overall cellular profile of immune cells within mucosal tissue was also assessed. The results from the characterization of tissue cells were compared to those seen in red blood cell-lysed whole blood. In addition, deconvolution immunofluorescent microscopy was used to identify the location of immune effector cells and their associated FcR within the three types of mucosal tissue.

FcR expression on immune effector cells isolated from all three mucosal tissue types was lower when compared to whole blood-derived immune effector cells. Where present, immune effector cells tended to be located within the subepithelium or basale stratum of the tissue (penile and ectocervical) or within the lamina propria (colorectal).

## Materials and Methods

### Tissue Samples

Ectocervical tissue (n = 5 CD14+ & CD11c FcR phentyping; n = 4 NK FcR phenotyping) was acquired from women undergoing planned therapeutic hysterectomy at St Mary’s Hospital (London, United Kingdom). Penile glans tissue (n = 9 CD14+ & CD11c FcR phentyping; n = 5 NK FcR phenotyping) was acquired from men undergoing elective gender reassignment surgery at Charing Cross Hospital (London, United Kingdom). Surgically resected specimens of colorectal tissue (n = 7 CD14+ & CD11c FcR phentyping; n = 3 NK FcR phenotyping) were collected at St Mary’s Hospital (London, United Kingdom) from patients undergoing rectocele repair and colectomy for colorectal cancer. Only healthy tissue obtained 10 to 15 cm away from any tumour was employed.

### Tissue Digestion

Tissue was cut into 3mm^3^ explant-sized pieces. A maximum of 5 of these pieces were further diced using a scalpel blade and then placed into 1mL of Liberase enzyme digestion cocktail (serum-free complete RPMI [1x penicillin/streptomycin solution (100 units, 0.1mg/mL, respectively), 2mM L-glutamate], 12.5μg/mL Liberase DL (colorectal tissue) or Liberase TL (penile or cervical tissue), 25μg/mL Hyaluronidase, 200μg/mL DNase) for either 1h (colorectal tissue) or up to 3h (penile or cervical tissue) at 37°C with 1200rpm shaking. After enzymatic digestion, the tissue was further mechanically disrupted and isolated cells were washed with complete RPMI containing 10% foetal calf serum, collected and passed through a 50μm nylon filter. Cells were stained with the flow cytometry panels immediately after isolation.

### Whole Blood Isolation and RBC-lysis

Whole blood samples were obtained from six HIV-negative donors. The samples underwent a red blood cell-lysis step before being stained with the flow cytometry panels. Briefly, 1x RBC-lysis buffer (Biolegend, UK) was made up from the 10x stock using deionized water, as per manufacturers’ instructions. 1mL of 1x RBC-lysis buffer was added to 10mL of freshly obtained whole blood. The blood was incubated in the dark for 5 minutes before the addition of 30mL of complete RPMI containing 10% foetal calf serum and subsequent centrifugation at 1500rpm for 5 minutes. These steps were repeated 3 times to ensure a RBC-free pellet. To assess the potential impact of digestion enzymes on cells, a mock digestion was performed using Liberase TL under the conditions for penile tissue, mentioned above.

### Flow Cytometry Staining

Red blood cell-lysed whole blood or cells isolated from mucosal tissues were stained using a combination of multicoloured flow cytometry panels designed to determine Fc-receptor expression on CD14+ monocytic cells, mDC or NK cells. Briefly, for overall cellular phenotyping; CD3 V450 [UCHT1], CD4 PECy7 [SK3] (Biolegend), CD8 Pacific Orange [3B5] (Invitrogen), CD19 BV650 [SJ25C1]. For CD14 and mDC FcR Phenotyping; CD3 V450 [UCHT1], CD14 Qdot 605 [TüK4] (Invitrogen), CD16 Pacific Orange [3G8] (Invitrogen), CD11c A700 [B-ly6], CD123 PECy5 [9F5], CD32 APC [FLI8.26], CD64 APC H7 [10.1], CD89 PE [A59], CD19 FITC [HIB19]. For NK cell FcR phenotyping; CD15 BV650 [W6D3] (Biolegend), CD16 PECy7 [3G8] (Biolegend), CD66b FITC [G10F5] (Biolegend), CD64 APC H7 [10.1], CD56 PECy5 [HCD56] (Biolegend), CD45 A700 [HI30] (Biolegend), CD89 PE [A59], CD3 V450 [UCHT1], CD32 APC [FLI8.26]. Unless otherwise specified, all antibodies were sourced from BD Biosciences. Anti-FcRs were able to detect antibody-occupied FcR as indicated by the manufacture and in house controls. Dead cells were excluded from analysis through staining with Aqua Viability Dye (Invitrogen).

### Flow Cytometry Acquisition and Analysis

Samples were acquired using a FACS LSRIIFortessa (BD Biosciences) and analysed using FlowJo (Tree Star, Ashland, OR, USA) and PESTLE and SPICE (National Institute of Allergy & Infectious Diseases, USA).

Compensation matrices were created on FlowJo using single stained anti-mouse Ig, κ/negative control compensation beads (BD Biosciences).

### Immunofluorescence and Imaging

Shortly after removal, all tissue samples were embedded in optimal cutting temperature (OCT) for longitudinal sectioning of epithelium and stored at -80°C until processing. Following, tissues were sectioned (12μm) and fixed in 3.7% formaldehyde in PIPES buffer and blocked with normal donkey serum prior to staining. To identify target cells, tissues were stained with a CD56 (BD Biosciences), CD11c (Invitrogen), or CD14 (Biolegend) antibody. Additionally, for Fc receptor identification, CD16 (AbD Serotec), CD32 (Abcam), CD64 (Abcam), or CD89 (LifeSpan Biosciences) antibodies were utilized. Secondary antibodies, Rhodamine RedX (Jackson ImmunoResearch) and Cy5 (Jackson ImmunoResearch), were also used. Antibody specificity was determined by negative results with respective isotype control antibodies. Hoechst DAPI (Invitrogen) was used for DNA staining. After staining, mounting medium (DakoCytomation) and coverslips were applied and sealed with nail polish. All images were obtained by deconvolution microscopy on a DeltaVision RT system collected on a digital camera (CoolSNAP HQ; Photometrics) using a 40x oil objective

### Statistical Analysis

Graphs show mean values with standard deviation error bars. Kruskal-Wallis test with Dunn’s multiple comparison test was used to compare the different tissues and WB samples. All statistical analyses were performed using Prism 6 (GraphPad Software, Inc. La Jolla, CA, USA).

### Ethics Statement

Written informed consent was obtained from all donors. All tissues were collected under protocols approved by the Imperial College NHS Trust Tissue Bank and the National Research Ethics Committee in accordance with the Human Tissue Act 2004. Approval for this project was granted by the Imperial College Healthcare Tissue Bank, under their HTA research licence, and ethics thus conveyed through this process by the Multi Research Ethics Committee (MREC), Wales.

## Results

### Characterization of immune cell phenotypes within mucosal tissue

Flow cytometry panels were designed to characterize CD3+ T-cells, CD14+ monocytic cells, CD19+ B-cells, myeloid dendritic cells (mDC), NK cells, NKT cells and neutrophil populations across different mucosal portals of infection, as well as RBC-lysed whole blood.

Cells were isolated from tissue using enzymatic digestion with Liberase DL (colorectal tissue) or TL (penile and cervical tissue). Enzyme concentrations were optimized and tested on whole blood under the same digestion conditions to ensure no degradation/loss of markers of interest (S1 Fig).

Similar percentages of NKT cells (CD3+CD56+), mDC and CD3+ T-cells were observed across the three mucosal tissue types and whole blood, although a trend towards higher expression of CD3+ T-cells was observed for cells isolated penile tissue.

**Fig 1:**
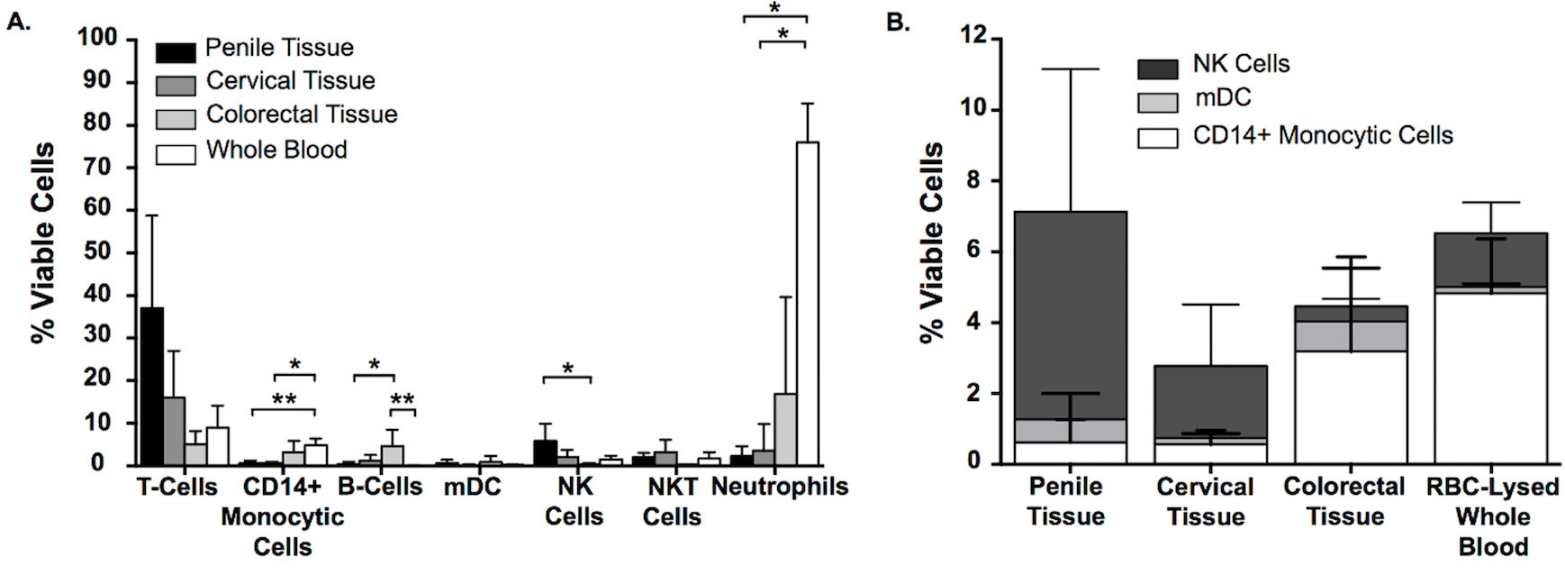
Characterization of immune cell phenotypes within mucosal tissue and RBC-lysed whole blood. The percentage of CD3+ T-Cells, CD14+ monocytic cells, CD19+ B cells, myeloid dendritic cells (mDC), NK cells, NKT cells and neutrophils in total viable cells isolated from (A) penile glans (n = 9), ectocervical (n = 5) and colorectal (n = 6) tissue and Whole Blood (n = 6). (B) Relative proportions of CD14+ monocytic cells, mDC and NK cells in penile, cervical and colorectal tissue and Whole Blood (mean and SD values shown).

CD14+ monocytic cell percentages were statistically higher for cells isolated from whole blood when compared to penile (P = 0.046) and cervical tissue (P = 0.037). A greater percentage of CD19+ B-cells were isolated from colorectal tissue when compared to penile tissue (P = 0.037) and whole blood (P = 0.029) (Fig 1A).

NK cells (CD3-CD56+) were noted at similar percentages in viable cells isolated from whole blood and cervical and penile tissue. There was a trend for higher percentages of NK cells isolated from penile tissue when compared to all other compartments, although this difference only reached statistical significance when compared to cells isolated from colorectal tissue (P = 0.011) (Fig 1A).

Neutrophils (CD3-CD15+CD66b+) were the most abundant cell subset isolated from whole blood, representing approximately 80% of all viable cells. When compared to the three tissue compartments, there were significantly higher percentages of neutrophils isolated from whole blood when compared to penile (P = 0.035) and cervical tissue (P = 0.011) (Fig 1A). It is important to note that neutrophils were found infrequently within all three mucosal tissues, with too few cell numbers acquired to perform meaningful Fc receptor expression analysis.

### CD14+ Fc Receptor (FcR) expression in whole blood and mucosal tissue compartments

**Fig 2:**
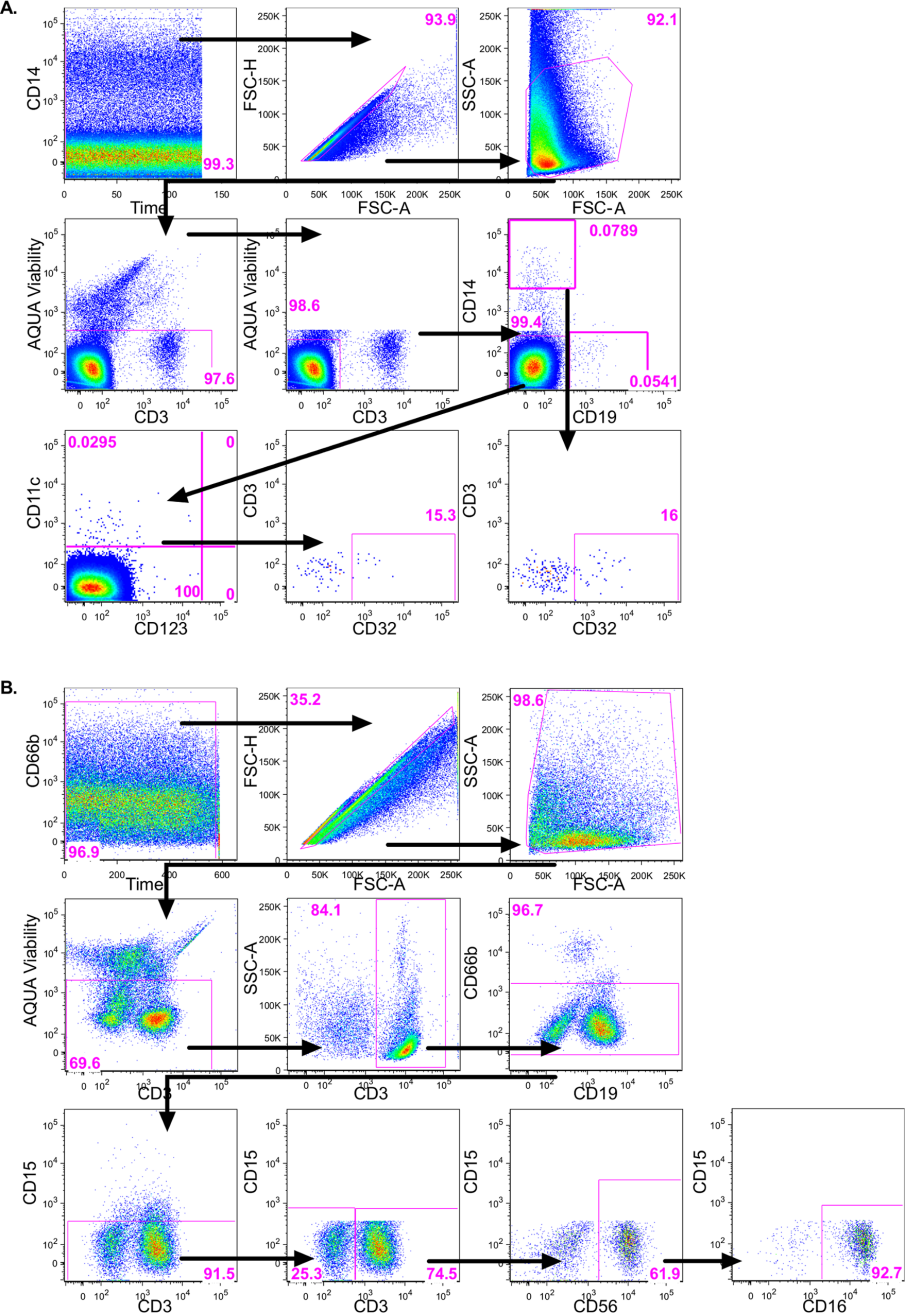
Flow cytometry analysis gating strategies in FlowJo. A time gate was initially applied to exclude any electronic noise followed by a singlet gate excluded any doublets, then a gate was applied to include the cells of interest, followed by a viability gate to exclude any dead cells. (A) FcR analysis for both CD14+ and mDC; CD3-negative cells were included and split into CD14+ or CD14-CD19- cells. CD14-CD19- were further categorized based on their CD11c expression. Finally, CD14+ cells and mDC were assessed for their FcR expression (only CD32 shown here). (B) NK FcR phenotypic analysis was assess by investigating the CD56+ cells for their FcR expression (only CD16 shown here). Representative plots for cells isolated from penile glans tissue.

CD14 is expressed on the surface of monocytic cells, mainly macrophages and dendritic cells, which are capable of phagocytosis of invading pathogens. To understand the FcR expression (CD16 [FcγRIII], CD32 [FcγRII], CD64 [FcγRI] and CD89 [FcαR]) on monocytic cell populations, CD14+ cells were defined using the gating strategy outlined in Fig 2A.

**Fig 3:**
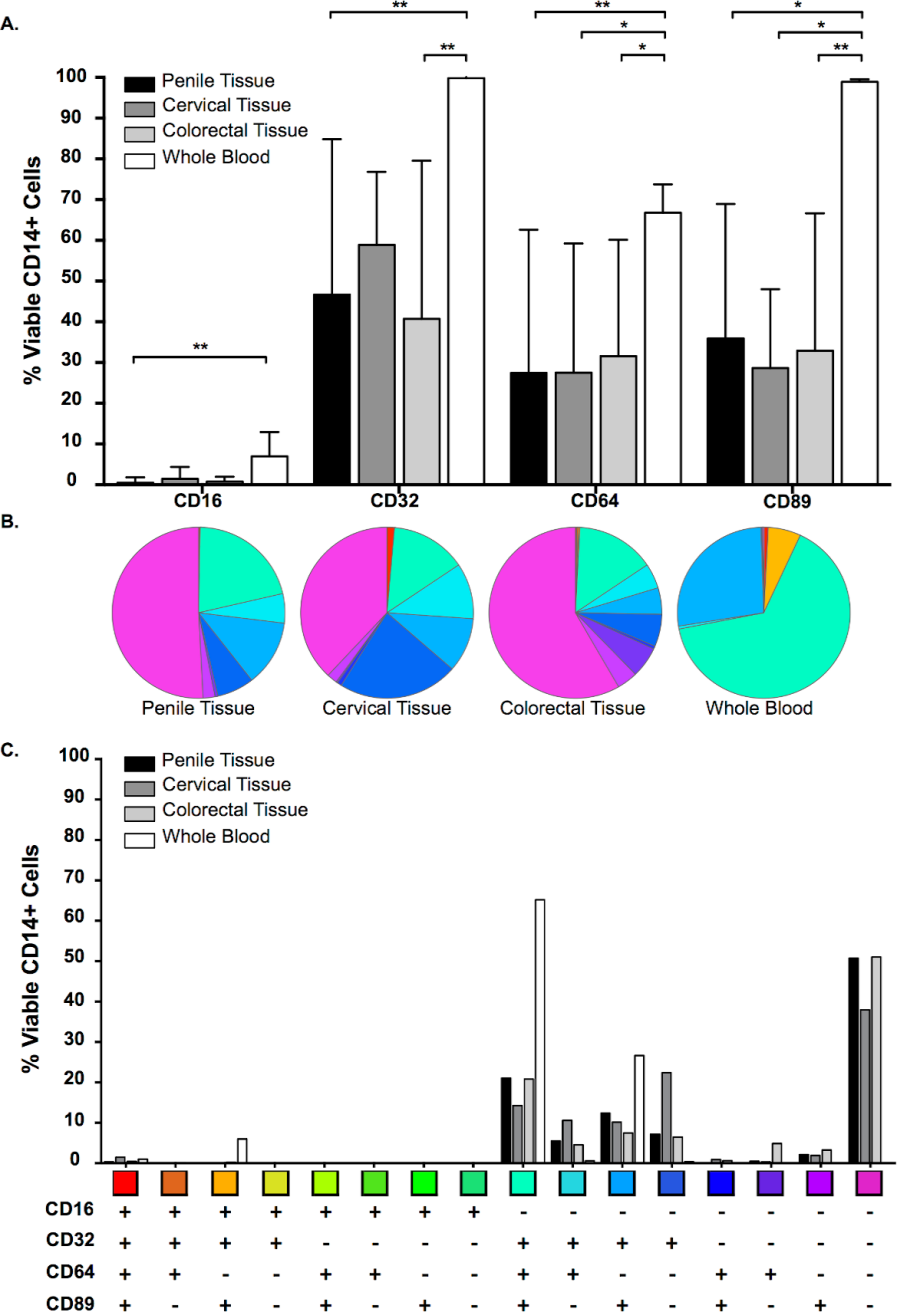
CD14+ Fc Receptor (FcR) expression in RBC-lysed whole blood and mucosal tissue compartments. Percentage expression of CD16, CD32, CD64 and CD89 on viable CD14+ cells (A) isolated from penile glans (n = 9); ectocervical (n = 4) and colorectal (n = 6) tissue and whole blood (n = 6) [Mean/SD values shown; comparisons of the FcR in the different tissue were made using Kruskal-Wallis with Dunn’s multiple comparison test]. (B) and (C) Boolean gating of FcR-positive cells to demonstrate the combinatorial nature of FcR expression in viable CD14+ cells across the three tissues and RBC-lysed whole blood (mean values shown).

CD16 was detected at higher levels on CD14+ cells isolated from whole blood when compared to cells isolated from all mucosal tissues, although this difference only reached significance when compared to penile tissue (P = 0.005) (Fig 3A). CD32 was found at higher levels on CD14+ monocytic cells isolated from whole blood when compared to penile (P = 0.005) and colorectal (P = 0.007) tissue, whereas the lower levels of expression noted on cells isolated from cervical tissue did not reach significance. Higher levels of CD64 and CD89 were detected on the surface of CD14+ cells isolated from whole blood when compared to penile (P = 0.007 & P = 0.014, respectively), cervical (P = 0.022 & P = 0.038, respectively) and colorectal tissue (P = 0.028 & P = 0.008, respectively) (Fig 3A).

Boolean gating was applied to investigate the combinatorial nature of the FcR expression. Approximately half of the viable CD14+ monocytic cells isolated from the three mucosal tissues were negative for all FcR when compared to whole blood, which showed co-expression of CD32, CD64 and CD89 in approximately 65% of CD14+ cells (Fig 3B & 3C). This phenotypic difference could be due to downregulation of FcR at the mucosal sites to prevent over-stimulation of the effector cells within the mucosal environment and/or reflect terminal differentiation.

Of the cells that were positive for FcR in all mucosal tissues, higher expression levels of receptors associated with ADCP and ADCVI (CD32, CD64 and CD89) were observed when compared to ADCC (CD16). Whilst it is important to note that there is no significant difference in the level of FcR expression between the three mucosal tissues, there were approximately 6-fold more CD14+ monocytic cells found in colorectal tissue when compared to penile and cervical tissue (Fig 1B). This observation potentially means that, while the percentage of CD14+ FcR+ cells do not differ between tissue types, there could be differences in the absolute number of cells capable of effector functions.

### Fc Receptor Expression on Myeloid Dendritic Cells

Myeloid dendritic cells (mDC) are involved in antigen presentation and phagocytosis of infectious pathogens. To study the frequency of FcR on the surface of these cells the mDC population was defined as any viable cells that were CD14^-^, CD19^-^ and CD11c^+^ (Fig 2A).

**Fig 4:**
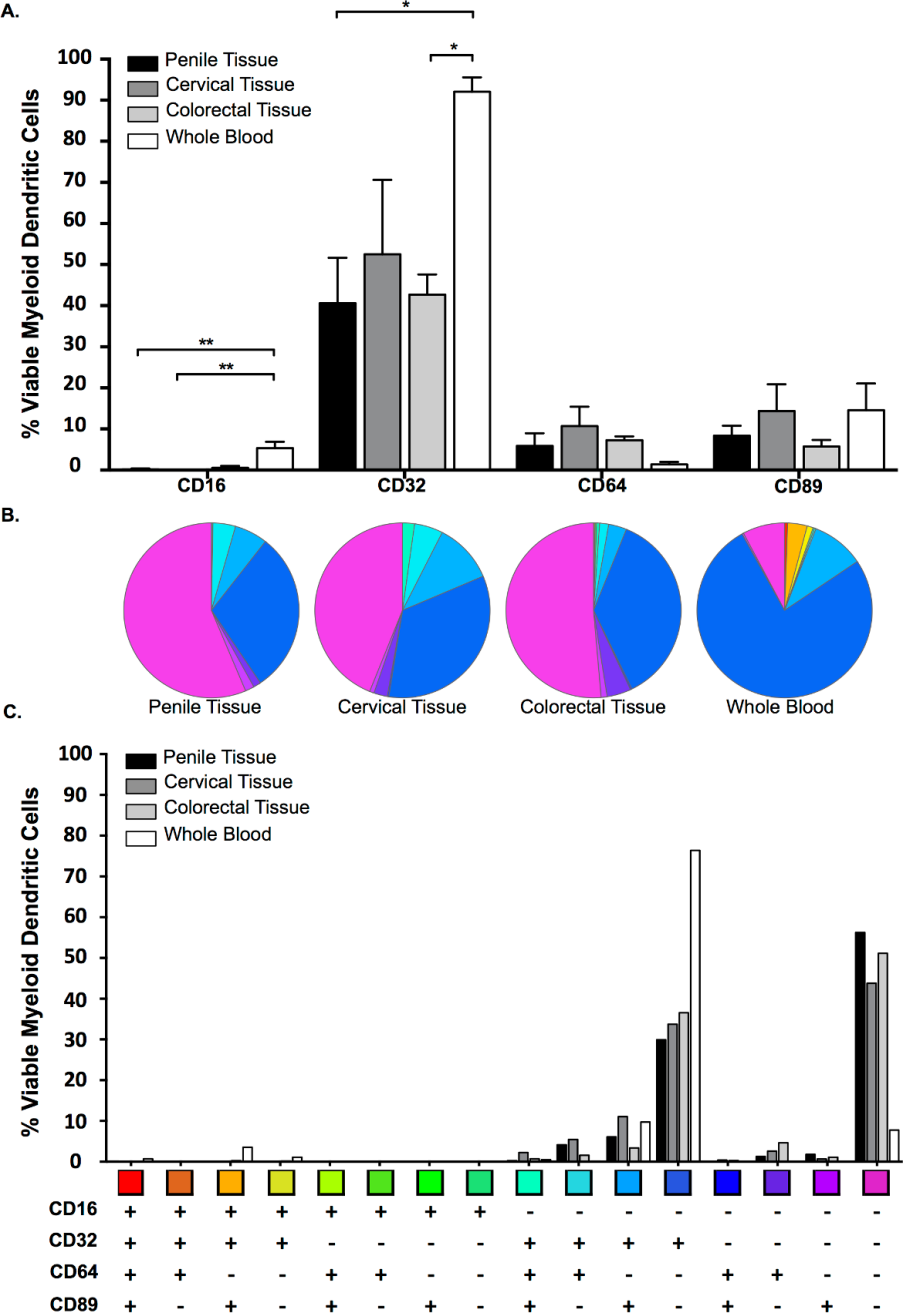
Myeloid Dendritic Cell Fc Receptor (FcR) expression in RBC-lysed whole blood and mucosal tissue compartments. Percentage expression of CD16, CD32, CD64 and CD89 on viable mDC (A) isolated from penile glans (n = 9); ectocervical (n = 4) and colorectal (n = 4) tissue and RBC-lysed whole blood (n = 6) [Mean/SD values shown; comparisons of the FcR in the different tissue were made using Kruskal-Wallis with Dunn’s multiple comparison test]. (B) and (C) Boolean gating of FcR-positive cells to demonstrate the combinatorial nature of FcR expression in viable mDC across the three tissues and RBC-lysed whole blood (mean values shown).

CD32 was the most predominant FcR detected on mDC isolated from mucosal cells and whole blood. mDC isolated from whole blood expressed low levels of all FcR, with the exception of CD32, where it was detected on approximately 90% of cells (Fig 4A) and at significantly higher levels when compared to colorectal (P = 0.036) and penile (P = 0.012) tissue. CD16 was found at higher levels on mDC isolated from whole blood when compared to all mucosal tissue types, reaching significance when compared to cervical (P = 0.004) and penile (P = 0.002) tissue. There was a trend for greater levels of CD64 on the surface of mDC isolated from all three mucosal tissues when compared to whole blood. However, the difference did not reach significance. Similar levels of CD89 were noted across all compartments (Fig 4A).

The proportionality of mDC FcR expression across different tissue showed a greater diversity of receptor expression within mucosal tissue when compared to whole blood, which almost exclusively expressed CD32 (Fig 4B & 4C). Boolean gating, used to determine the complexity of FcR combinatorial expression within mDC, indicated that, while a greater proportion of mDC isolated from mucosal tissue did not express any FcR when compared to the whole blood, the mDC that did were more diverse in their expression (Fig 4B & 4C). Although there was no significant difference between the percentage of mDC found in the three mucosal tissues, there was a trend for increased percentages in penile and colorectal tissue (Fig 1B). This observation could potentially impact on the absolute numbers of cells available for Fc-mediated effector functions.

### NK Cell Fc Receptor (FcR) expression in whole blood and mucosal tissue compartments

Natural Killer cells (NK) are cytotoxic cells involved in the killing of virally infected cells through release of cytokines. FcR play an important role in bridging the gap between these innate cells and antibodies. To study the frequency of FcR on the surface of these cells the NK cell population was defined as any viable cells that were CD3^-^, CD56^+^ (Fig 2B).

**Fig 5:**
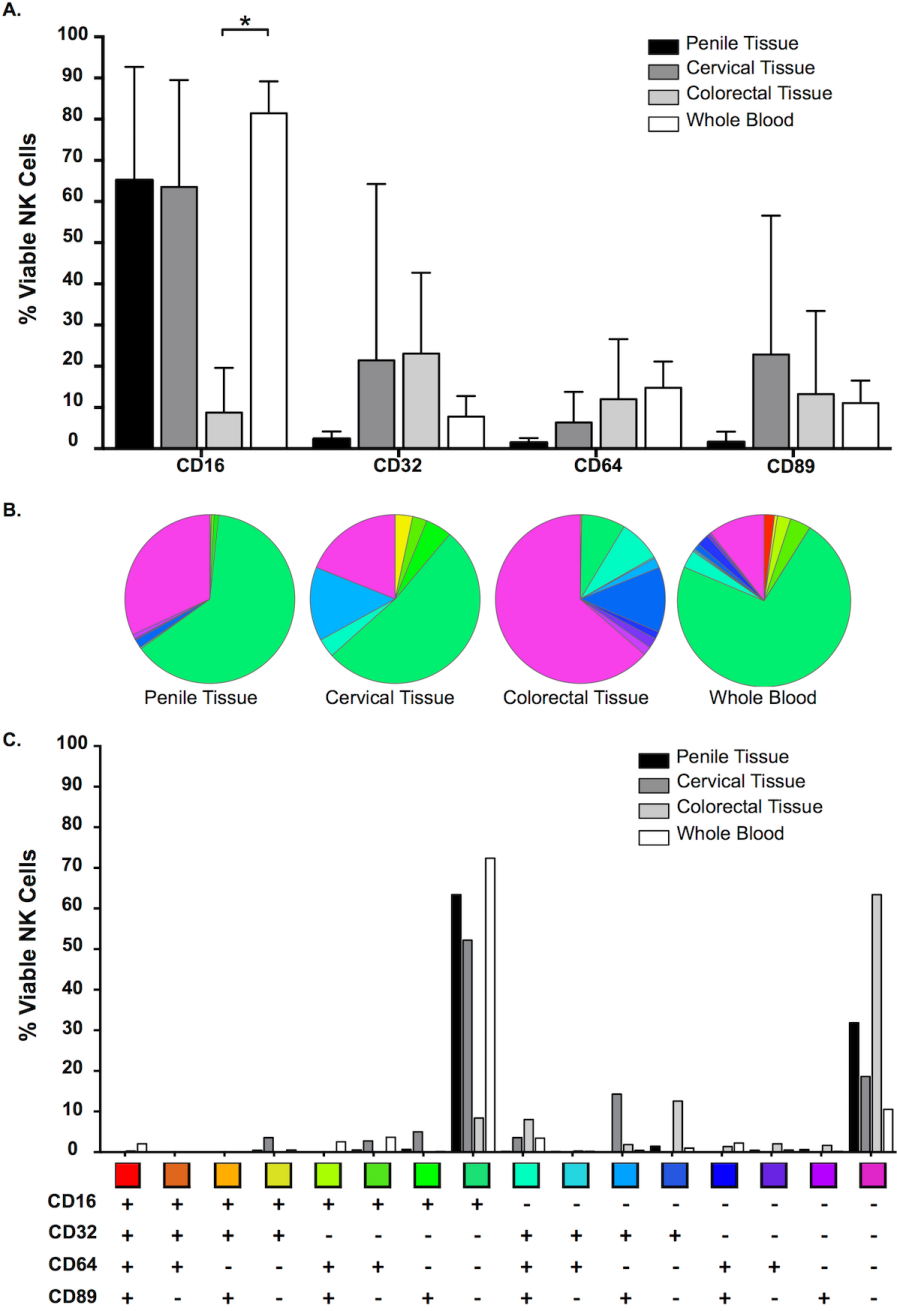
NK Cell Fc Receptor (FcR) expression in RBC-lysed whole blood and mucosal tissue compartments. Percentage expression of CD16, CD32, CD64 and CD89 on viable NK (A) cells isolated from penile glans (n = 5); ectocervical (n = 4) and colorectal (n = 3) tissue and RBC-lysed whole blood (n = 6) [Mean/SD values shown; comparisons of the FcR in the different tissue were made using Kruskal-Wallis with Dunn’s multiple comparison test]. (B) and (C) Boolean gating of FcR-positive cells to demonstrate the combinatorial nature of FcR expression in viable NK cells across the three tissues and RBC-lysed whole blood (mean values shown).

CD16 was the predominant FcR detected on NK cells isolated from cervical and penile tissue and whole blood. Whilst there were fewer CD16+ NK cells isolated from colorectal tissue when compared to the other compartments, this difference only reached significance when compared to NK cells isolated from whole blood (P = 0.033). CD32, CD64 and CD89 were seen at similar levels on NK cells isolated from cervical and colorectal tissue and whole blood, with lower levels noted on penile tissue. However, this difference did not reach significance (Fig 5A).

Boolean gating analysis to assess the combinatorial expression of FcR on NK cells demonstrated that there were more FcR negative NK cells isolated from mucosal tissue when compared to whole blood. This phenotypic difference was most evident for NK cells isolated from colorectal tissue, which included approximately 65% FcR negative cells. Cells isolated from cervical tissue were the most diverse in their FcR phenotype expression (Fig 5B & 5C).

A trend for a higher percentage of NK cells was noted in the penile tissue, reaching significance when compared to the colorectal tissue (Fig 1B). Whilst this increased percentage does not give details as to absolute numbers of NK cells present, it is important to remember that it could impact on the number of FcR and effector cells available for further downstream functions.

### Location of immune effector cells and Fc Receptors within mucosal tissue

To better understand the availability of immune effector cells within mucosal tissue, we used deconvolution microscopy to determine the location of CD14+, CD11c+ or CD56+ cells within all three mucosal tissue types. Secondary staining was used to further assess the FcR expression within the tissues.

**Fig 6:**
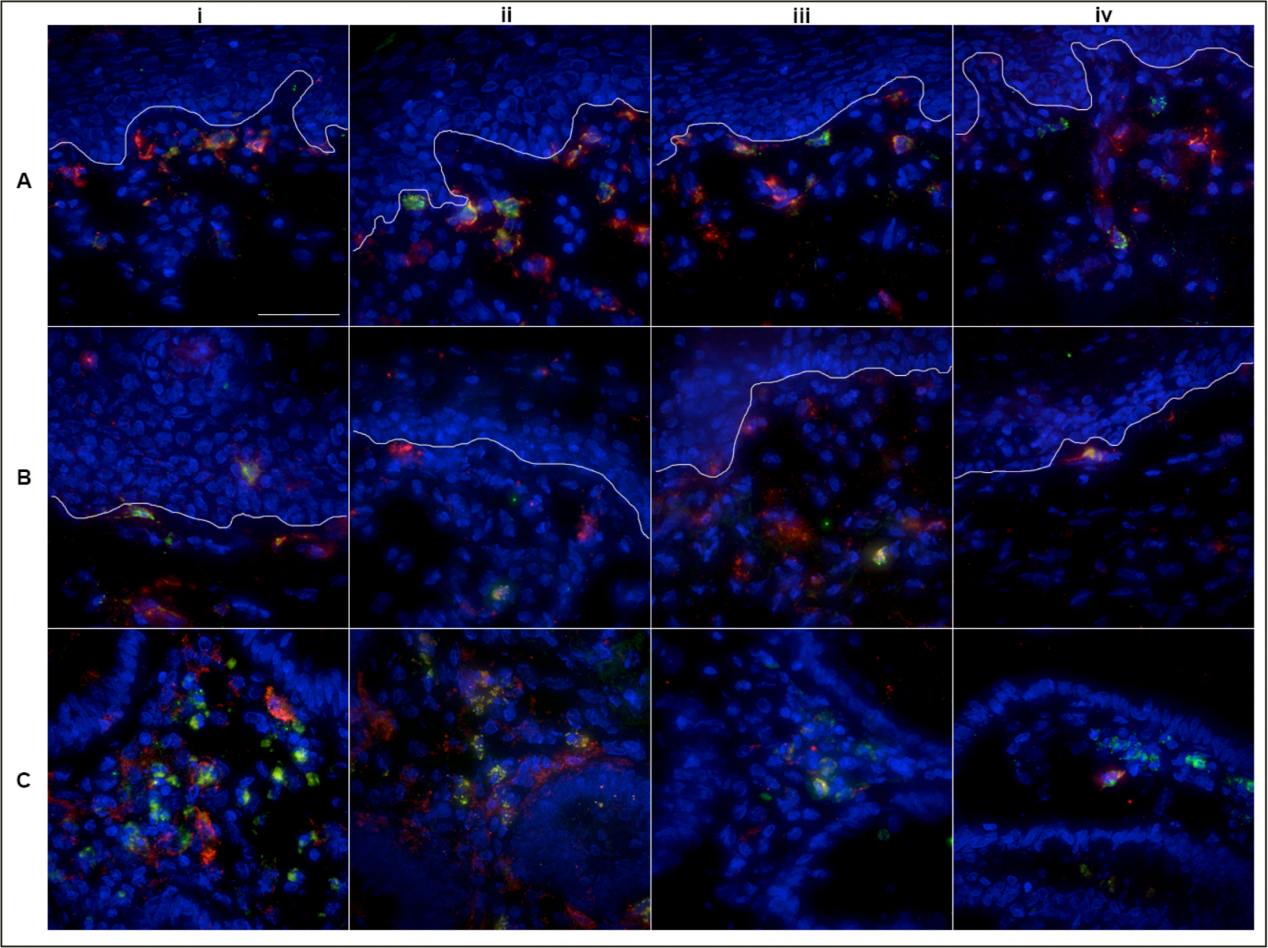
Localization of CD14+ cells within three different mucosal tissue types. Deconvolution microscopy images showing location of CD14+ cells within Penile Glans (A), Ectocervical (B) and Colorectal tissue (C) and relative co-expression of FcR CD16 (i), CD32 (ii), CD64 (iii) and CD89 (iv). CD14 is shown in green, the FcR are shown in red and DAPI is shown in blue. Images taken at 40x; Scale bar set to 40 microns.

Within the squamous epithelium of the penile glans, CD14+ cells were almost exclusively located in the subepithelial lamina propria. In one, rare instance, a CD14+ cell was noted within the stratum basale (Fig 6A). Within the ectocervical tissue, CD14+ cells were found to reside within the subepithelium and stratum basale (Fig 6B) whereas within the colorectal tissue, CD14+ cells were exclusively found within the lamina propria (Fig 6C).

**Fig 7:**
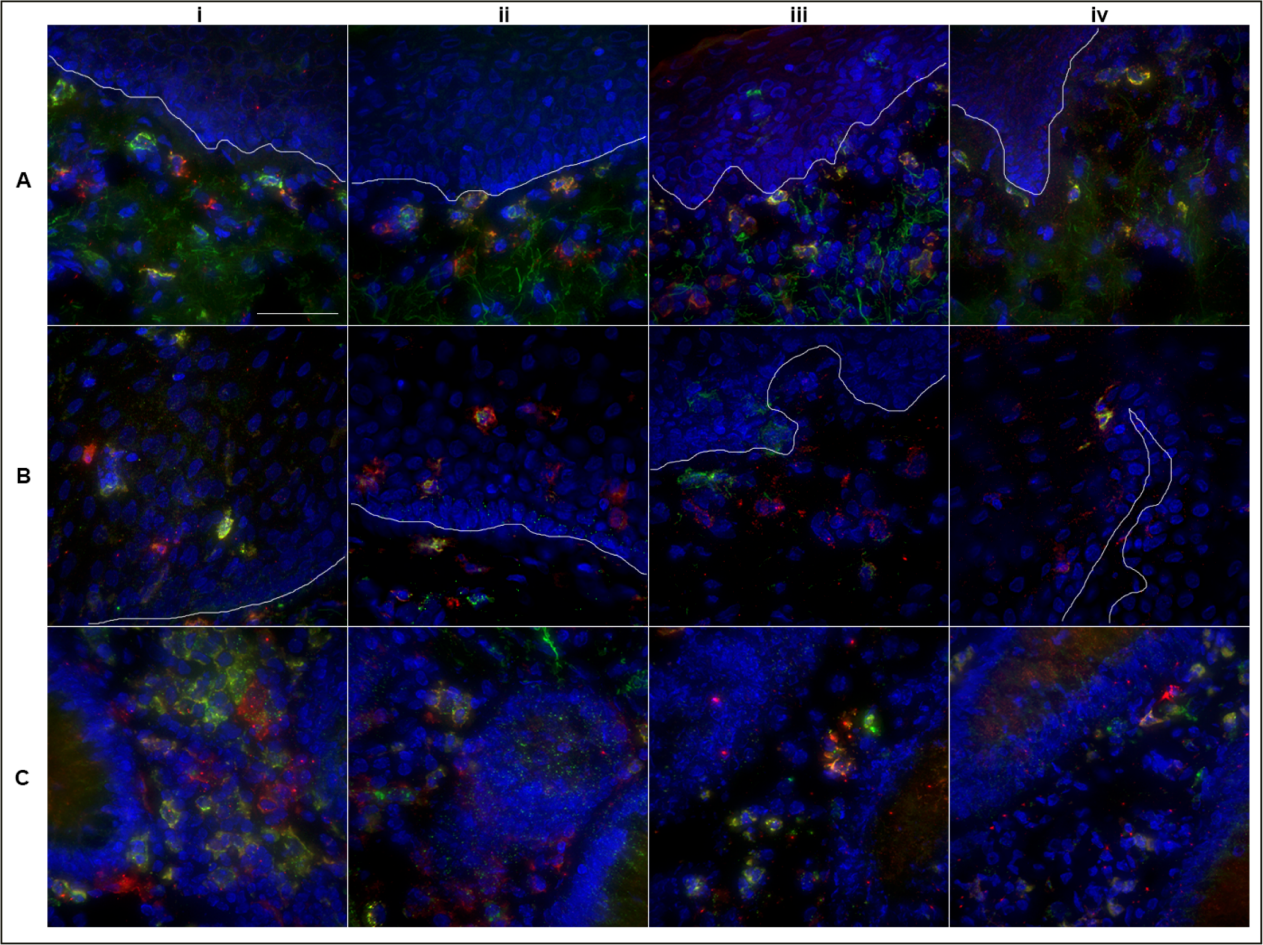
Localization of CD11c+ cells within three different mucosal tissue types. Deconvolution microscopy images showing location of CD11c+ cells within Penile Glans (A), Ectocervical (B) and Colorectal tissue (C) and relative co-expression of FcR CD16 (i), CD32 (ii), CD64 (iii) and CD89 (iv). CD11c is shown in green, the FcR are shown in red and DAPI is shown in blue. Images taken at 40x; Scale bar set to 40 microns.

CD11c+ cells were exclusively found within the subepithelial lamina propria of the penile tissue (Fig 7A), whilst they were more diffusely located throughout the squamous epithelium of the cervical tissue, from the stratum spinosum into the stratum basale and deeper into the subepithelium (Fig 7B). CD11c+ cells of the colorectal tissue were found to reside within the lamina propria only (Fig 7C).

**Fig 8:**
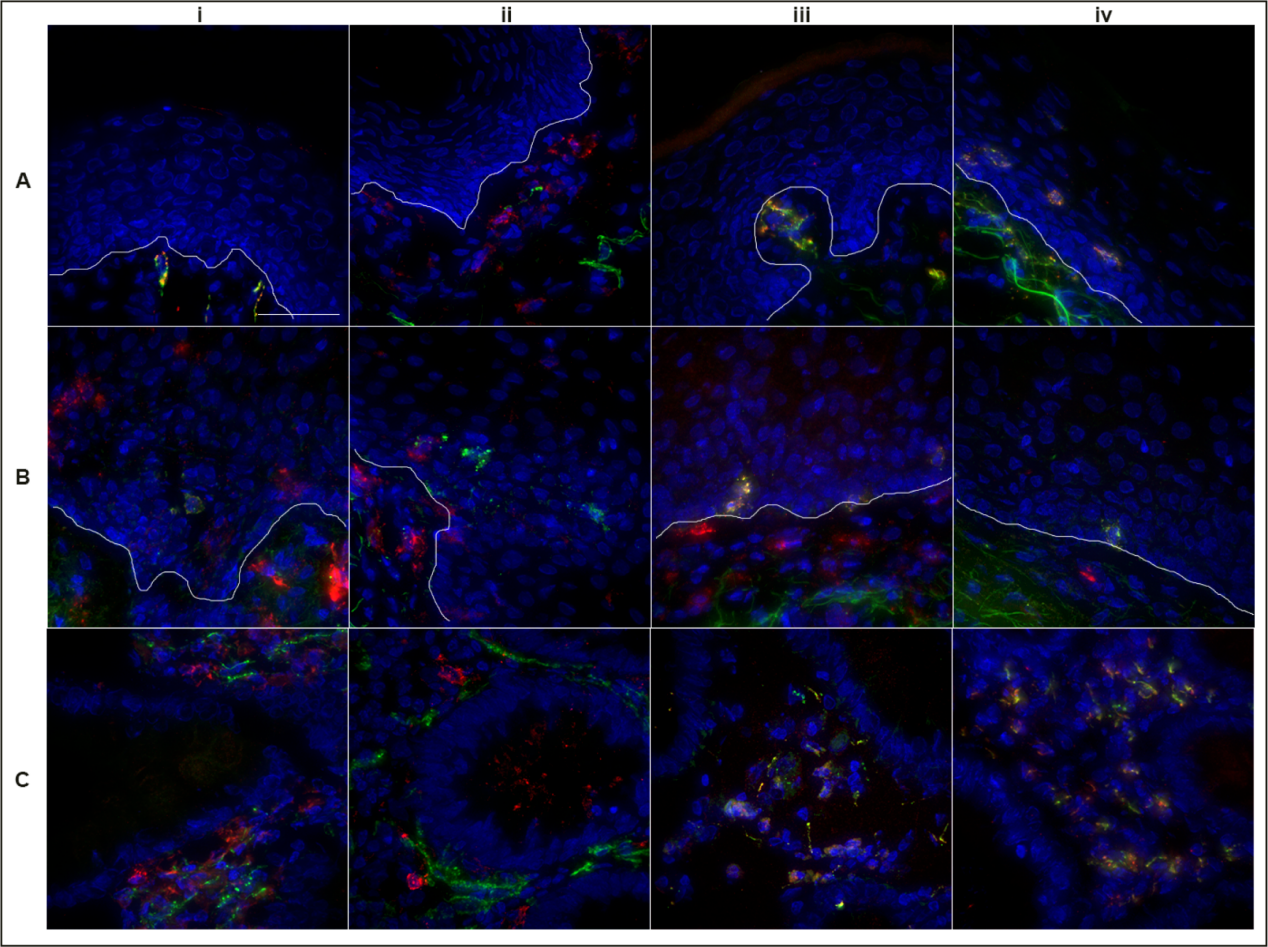
Localization of CD56+ cells within three different mucosal tissue types. Deconvolution microscopy images showing location of CD56+ cells within Penile Glans (A), Ectocervical (B) and Colorectal tissue (C) and relative co-expression of FcR CD16 (i), CD32 (ii), CD64 (iii) and CD89 (iv). CD56 is shown in green, the FcR are shown in red and DAPI is shown in blue. Images taken at 40x; Scale bar set to 40 microns.

CD56+ cells of the penile tissue were the most diffusely located effector cell type that we stained for within this tissue. Whilst the majority resided within the subepithelial lamina propria, we also observed CD56+ cells within the stratum basale and, on one occasion, a CD56+ cell was noted near the luminal border (Fig 8A). Within cervical tissue, CD56+ cells were primarily observed within the subepithelium or stratum basale (Fig 8B), whereas CD56+ cells of the colorectal tissue were, once again, found to be exclusively located within the lamina propria (Fig 8C). Across all mucosal tissues, co-localization of the effector cells and FcR were in line with the flow cytometry data (Figs 6–8 & S2–S4 Figs).

## Discussion

To better understand the potential of antibody-dependent cellular effector functions to impact on initial events in mucosal HIV-1 transmission, we characterized the relative FcR expression levels and composition of FcR positive cells within male and female genital mucosal tissue, colorectal tissue and RBC-lysed whole blood.

We observed similar percentages of CD3+ T-cells, NKT cells and mDC across the three tissue types and whole blood. However, there were significant differences between the percentages of CD14+ cells, CD19+ cells and neutrophils between tissues and whole blood.

Colorectal tissue is known to contain large numbers of B cells and is in keeping with the observed higher numbers of CD19+ cells isolated from the colorectal tissue compared to cervical and penile glans tissue [27, 28]. Previously, it has been shown that macrophages within intestinal and, more specifically, colonic tissue have low levels of CD14 [25, 29]. Nevertheless, we show that the frequency of CD14+ cells was higher in cells isolated from colorectal tissue when compared to penile and cervical cells, albeit at lower levels when compared to those found in isolated RBC-lysed whole blood. It is important to note that these previous studies focused on pure macrophage populations isolated from intestinal tissue, whereas we have performed analyses on all CD14+ cells from a total viable cell population isolated from mucosal tissue, with no further markers to absolutely confirm that these cells are of a macrophage phenotype. More recently, another study used confocal microscopy to show levels of CD14+ cells in sigmoid colon and rectal mucosa are more in line with the results presented here [30]. Gut tissue-resident macrophages are almost exclusively derived from the recruitment of blood monocytes [31, 32] and it is therefore possible that the higher frequency of CD14+ cells observed in colorectal tissue when compared to the two other mucosal tissues (cervical and penile glans) investigated is linked to a higher influx of these cells from the circulation.

In line with previous studies, our data show that neutrophils are the major leucocytes found within whole blood [33], yet a minor cell subset within skin (penile glans) [34], ectocervical [35] and colorectal tissue [36]. Indeed, we isolated so few neutrophils from mucosal tissues that it was not possible to accurately determine the FcR expression on the surface of these cells. Neutrophils isolated from whole blood have been shown to constitutively express CD16b and CD89 at high levels and CD16a and CD32 at low levels [37]. CD64 can be induced to express on the surface of neutrophils leading to increased ADCC activity [38]. Given that neutrophils are commonly recruited during inflammation, it is possible that this cell subset could still play an important role in vaccine-induced immunity to HIV-1.

As previously determined, where immune effector cells were found, they tended to be located in the sub-epithelial and basal layers of the mucosal tissue [26, 28, 39, 40]. This could be of relevance when considering the accessibility of these cells to interact with vaccine-induced HIV-1 antibodies and prevent early viral transmission events.

Downstream effector functions during Fc-FcR activation are dependent upon the FcR activated and effector cell phenotype. The CD16 receptor, FcγRIII, is the intermediate affinity receptor for monomeric IgG and is most commonly associated with NK cell-mediated ADCC activity including release of lytic vesicles containing perforin and granzyme, but also with the induction of phagocytosis of immune complexes and cytokine release. CD32, FcγRII, is a low affinity IgG Fc-receptor that requires antibodies to have formed immune complexes with the antigen to trigger further downstream effects such as target cell lysis and/or phagocytosis. CD64, FcγRI, is the high affinity IgG Fc-receptor, which has particular affinity for IgG1 and IgG3 [41]. Lastly CD89, FcαR, is the IgA receptor, binding aggregated immunoglobulin A complexed with antigen leading to phagocytosis and ADCC [42]. FcγR have been implicated in the regulation of DC activity and are thought to play an integral role in determining whether inflammatory or tolerogenic responses are initiated during DC antigen presentation to T cell subsets [43].

CD16, commonly associated with ADCC, was observed at high levels on NK cells isolated from penile and cervical tissue and whole blood, but was low in colorectal tissue, in line with a previous study, which identified NK cells within normal colorectal tissue to be of a CD56+ CD16l^o/-^ phenotype [44]. Low levels of CD16 expression were detected within CD14+ cells, whereas the receptors more closely associated with the induction of ADCP and ADCVI (CD32, CD64 and CD89) were seen at comparatively higher levels. These data suggest ADCP and ADVCI might have increased potential over ADCC for impacting on HIV-1 transmission events.

CD32 is thought to be the most broadly expressed FcγR on immune effector cells found in the blood [45]. Results here support this statement and indicate that it is also the most prolific FcγR expressed across all three mucosal tissue types investigated, with data showing high expression levels on both CD14+ cells and mDC. CD32 is expressed as both inhibitory (CD32B) or activating (CD32A or CD32C) forms. These are capable of modulating the immune response depending on the presence or absence of co-stimulatory signals, such as engagement of CD40 or Toll-like receptors, which will promote an inflammatory response in DC [41]. Although this study was not designed to distinguish between these CD32 receptor phenotypes, their expression across different mucosal tissue types would be important to classify in future studies. Interestingly, recent studies have shown that antibodies from HIV-1 controllers and untreated progressors exhibit increased phagocytic activity, altered Fc domain glycosylation, and skewed interactions with CD32a and CD32b in both bulk plasma and HIV-specific IgG [18]. These data suggest specificity of vaccine-induced antibodies for CD32a over CD32b may be important for harnessing mucosal antibody-dependent effector function.

We show significantly lower levels of CD64 on the surface of CD14+ monocytic cells isolated from all three mucosal tissues when compared with freshly isolated whole blood. It is known that loss of CD64 on cell surfaces is an early indicator of myeloid cell maturation [46]. This observation likely reflects that the CD14+ cell population found within mucosal tissue is of a more mature phenotype than that found within whole blood and could be important when considering the ease of inducing CD64-specific effector immune responses at mucosal surfaces through vaccination.

CD89 expression was found at low levels on the surface on all NK cells, irrespective of their origin. On the surface of CD14+ monocytic cells and mDC, CD89 was found at comparatively similar levels in all three mucosal tissue types investigated but, for CD14+ monocytic cells, at significantly lower levels when compared to the same cell types isolated from whole blood. This observation is similar to previous studies looking at CD89 expression in tissues, including gastrointestinal and skin [47].

FcR expression on monocytes, mDC, NK cells and neutrophils are regulated by numerous factors. Cytokines such as IL-4 and IFN-γ have been shown to upregulate the expression of FcγR on monocytes and PMN [48, 49] and TNF-α expression can upregulate FcγR and CD89 expression on NK cells [50, 51]. Conversely, it has been demonstrated that IL-10 downregulates FcγR expression on monocytes [52]. Cytokine production in a mucosal environment, particularly within the gastrointestinal tract, can be influenced by factors such as diet, pre/probiotics and antibiotics [53, 54]. Cyclical hormonal changes within the female reproductive tract also alter the cytokine milieu [55, 56]. For penile tissue, differences in cytokine production have been noted between inner & outer foreskin and the penile glans [57] and additional studies have investigated cytokine profiles in penile foreskin in healthy and HIV-1 infected individuals [58–60]. Furthermore, other sexually transmitted infections are highly likely to influence cytokine profiles and FcR expression. In broader terms, studies performed in skin also indicate that cytokine profiles differ within this tissue when compared to the periphery [61]. It should therefore be considered that the lower basal expression levels, but higher diversity of FcR seen in the three mucosal tissue tested in this study are likely as a result of differences in the local environment when compared to effector cells isolated from whole blood.

Additionally, it is important to note that the mucosal tissues sampled in this study were all obtained from patients attending clinics in London, UK. It is possible that FcR expression profiles could differ in individuals living in other parts of the world where there are additional external influences. For example, studies have demonstrated that *Plasmodium falciparum* infection induces upregulation of FcγR on monocytes and subsets of DC [62, 63]. These environmental factors could be relevant when considering potential mucosal FcR-dependent responses to HIV-1 vaccine trials initiated in malaria endemic areas. The data presented here serve to provide an important baseline that will facilitate the broader study of mucosal FcR expression in populations at high risk for HIV-1 infection.

Together, the results show that immune cells of mucosal tissue are phenotypically different when compared to those found in the periphery, which may have important implications for the potential of antibody-dependent cellular effector mechanisms to impact on HIV-1 transmission events. To date, most *in vitro* ADCC assays use blood-derived NK cells, neutrophil, monocytes or macrophages. While blood-derived monocytes mediate similar levels of ADCC to NK cells [64], it is unclear as to the relative potency of their mucosal counterparts. Indeed, our studies demonstrate that a large proportion of mucosal myeloid cells are negative for all FcR. Furthermore, mucosal myeloid cells positive for FcR displayed a greater diversity of FcR expression than those in the periphery, which presented more uniform levels of expression within any one population. Such diversity adds additional complexity to potential tailoring of vaccine-induced humoral responses to promote enhanced antibody-dependent effector cell function.

In summary, we have characterized the pattern of Fc-receptor expression on CD14+ monocytic cells, mDC and NK cells across three different mucosal tissues associated with HIV-1 transmission. Overall, FcR expression levels were significantly lower in effector cells isolated from mucosal tissues than whole blood. The potential implication of the observed FcR expression patterns on the effectiveness of FcR-dependent cellular effector functions to impact on the initial events in mucosal transmission and dissemination warrants further mechanistic studies. To address these issues, inhibition of virus replication by antibody-dependent effector mechanisms in mucosal tissues *ex vivo* is currently being explored.

## Supporting Information

S1 Data Raw data for Figs 1–5 within this manuscript. (XLSX)

S1 Fig A & B. Optimization of Liberase digestion in RBC-lysed whole blood.
(TIFF)

S2 Fig A & B. Immunofluorescent imaging of CD14+ cells within penile glans, ectocervical and colorectal tissue. Unmerged images shown.
(TIFF)

S3 Fig A & B. Immunofluorescent imaging of CD11c+ cells within penile glans, ectocervical and colorectal tissue. Unmerged images shown.
(TIFF)

S4 Fig A & B. Immunofluorescent imaging of CD56+ cells within penile glans, ectocervical and colorectal tissue. Unmerged images shown.
(TIFF)
