# Supplementary figures and images for: Expression Profile of Human Fc Receptors in Mucosal Tissue: Implications for Antibody-Dependent Cellular Effector Functions Targeting HIV-1 Transmission
Source: PLoS One. 2016 May 10;11(5):e0154656. doi: 10.1371/journal.pone.0154656 (PMC4862624; doi:10.1371/journal.pone.0154656)

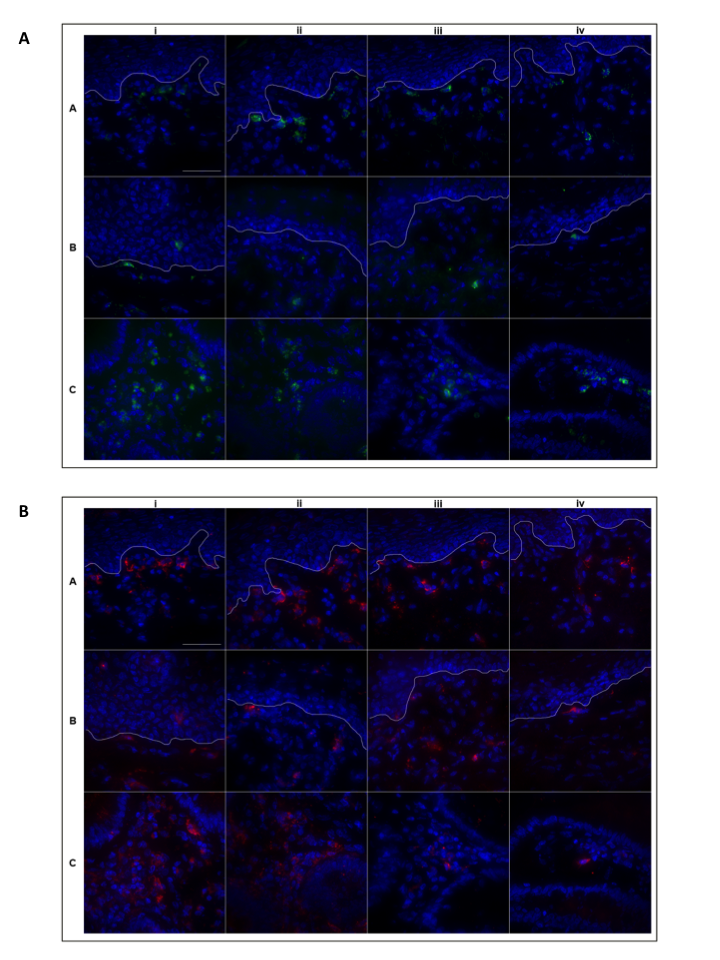

Supplement: S2 Fig — A & B. Immunofluorescent imaging of CD14+ cells within penile glans, ectocervical and colorectal tissue. Unmerged images shown. (TIFF) [file pone.0154656.s003.tiff]

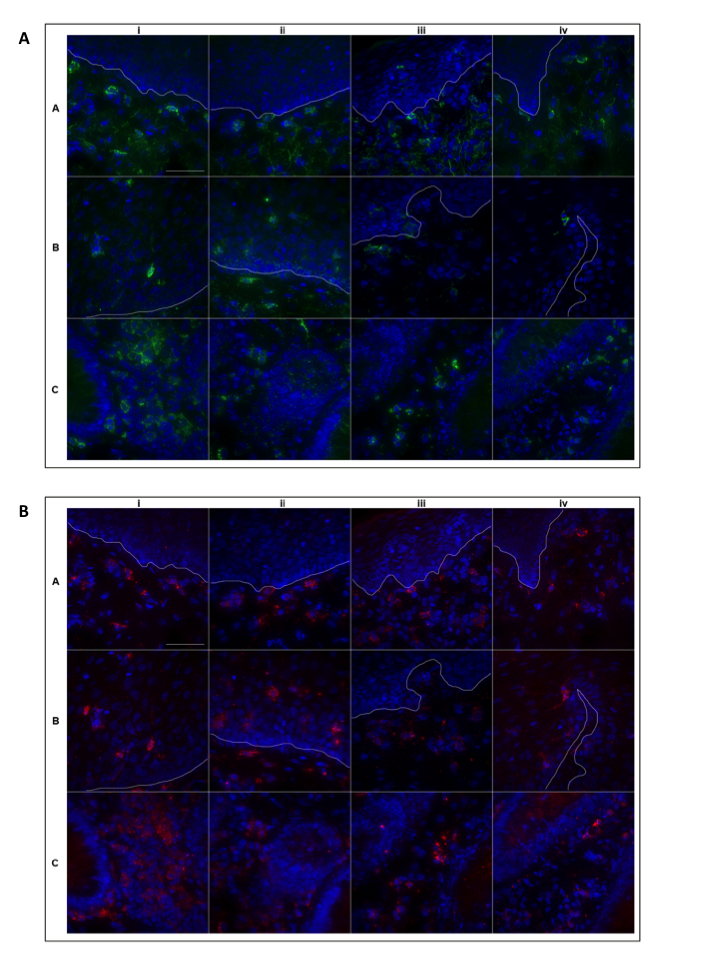

Supplement: S3 Fig — A & B. Immunofluorescent imaging of CD11c+ cells within penile glans, ectocervical and colorectal tissue. Unmerged images shown. (TIFF) [file pone.0154656.s004.tiff]

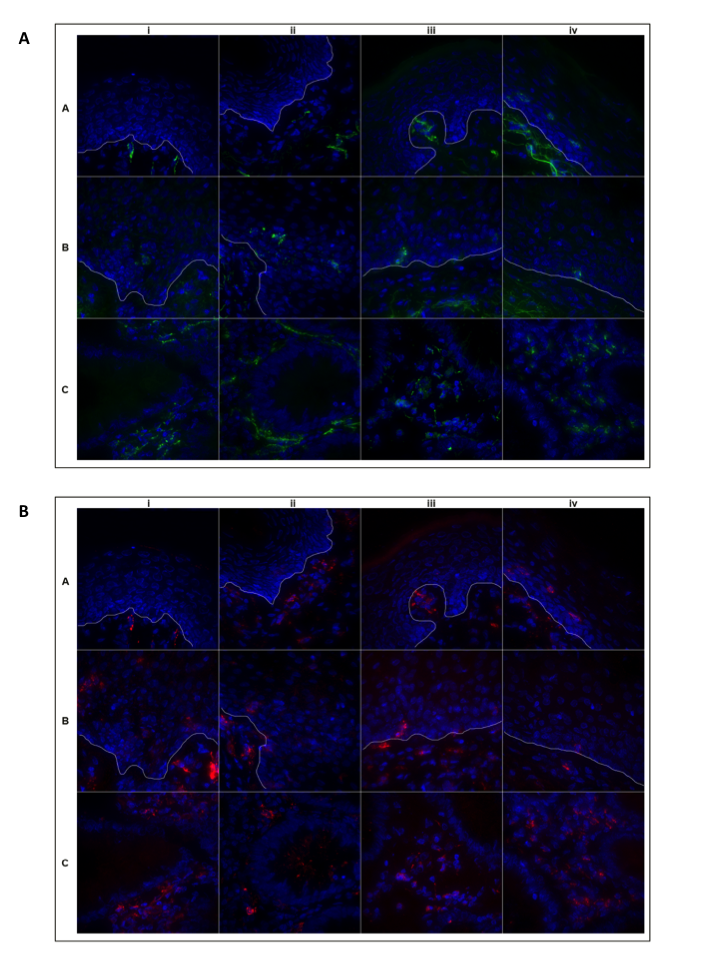

Supplement: S4 Fig — A & B. Immunofluorescent imaging of CD56+ cells within penile glans, ectocervical and colorectal tissue. Unmerged images shown. (TIFF) [file pone.0154656.s005.tiff]
